# Development and psychometric validation of a multidimensional institutional trustworthiness scale

**DOI:** 10.1017/cts.2026.10793

**Published:** 2026-07-13

**Authors:** Alena Kuhlemeier, Yu-Yu Hsiao, Mark L. Wieland, Irene G. Sia, Sagar Dugani, Nina Wallerstein, Prajakta Adsul

**Affiliations:** 1 College of Population Health, University of New Mexico Health Sciences Center, Albuquerque, NM, USA; 2 Department of Individual, Family, and Community Education, College of Education and Human Sciences, University of New Mexico, Albuquerque, NM, USA; 3 Community Internal Medicine, Mayo Clinic, Rochester, MN, USA; 4 Mayo Clinic, Rochester, MN, USA; 5 Center for Participatory Research, University of New Mexico Health Sciences Center, Albuquerque, NM, USA; 6 Medicine, Department of Internal Medicine, School of Medicine, University of New Mexico Health Sciences Center, Albuquerque, NM, USA

**Keywords:** Trustworthiness, community-engaged research, institutional context, contextual measurement

## Abstract

**Introduction:**

An academic medical center’s trustworthiness is a necessary precondition of patient and community trust. Current operationalizations of trustworthiness fail to capture the construct’s complexity at the institutional level. We propose a novel multidimensional scale to assess perceived trustworthiness of academic medical centers.

**Methods:**

We propose and psychometrically evaluate a 20-item Multidimensional Institutional Trustworthiness Scale (MITS). Invitations to complete an online survey in REDCap were sent to 175 leaders, investigators, staff and community partners affiliated with an academic medical center. Item-total correlations were calculated and construct validity assessed using confirmatory factor analysis.

**Results:**

Items in the hypothesized four factors demonstrated high factor loadings (>0.7) and explained substantial item variance (>50%), providing strong empirical support for the proposed scale and affirming its conceptual structure and measurement integrity. Tests of scale reliability showed high Cronbach’s alphas (>0.9) for all four factors, suggesting that all subscales produce consistent and dependable scores.

**Conclusions:**

This study provides support for the construct validity and scale reliability of a novel institutional trustworthiness scale, based on perspectives from community and academic affiliates of an academic medical center. We discuss the importance of measuring institutional trustworthiness using multiple dimensions to inform future interventions aiming to improve institutional trustworthiness.

## Introduction

Since the mid-20^th^ century, trust in healthcare and health research institutions in the United States has eroded significantly. While polls find that Americans have relatively high confidence in individual clinicians [1], they report very little confidence in the healthcare system [2]. After a substantial increase in reported trust in clinicians and the healthcare system in the United States associated with the COVID-19 pandemic [2,3], trust in the healthcare system has returned to pre-pandemic levels [2]. While single-item measures of trust administered in large, national polls can demonstrate the gravity of the problem of trust in healthcare, they are not sufficient to understand how to address this problem [4].

Research in this area points to two different manifestations of the problem of trust in healthcare: distrust and mistrust. Mistrust refers to a more individualized phenomenon wherein an individual believes that someone (or an institution) will not act in their best interests [5,6]. Often distrust is rooted in and/or exacerbated by misinformation. Mistrust, however, is more diffuse. It is rooted in historical experiences of oppression and less focused on a particular provider or institution [5].

The COVID-19 pandemic exposed the degree to which communities’ mistrust in the healthcare system can be traced back to the root causes of health inequities. Marginalized communities that have received suboptimal or inadequate healthcare report a higher level of mistrust in the healthcare system [7,8]. Historical abuses have also generated lack of trust in research and research institutions. Overall, lack of trust in research institutions has grown, with a January 2025 poll reporting that only 66% of the population trust the National Institutes of Health, with fewer, 61%, trusting recommendations from the Centers for Disease Control and Prevention [9].

Gray and colleagues proposed that “community engagement presents an opportunity for reckoning with the mistrust our profession has duly earned” [8]. While trust has been studied extensively at the level of individual research collaborations [10], the opportunity has grown for institutions to benefit from community engagement and more comprehensive measurement of those institutional practices that inspire trust. Addressing historic and systemic breaches in trust will require concerted, multi-faceted effort by healthcare systems and health research institutions to understand contemporary practices that undermine community trust. We conceive of trustworthiness of an institution as being comprised of a collection of beliefs a population has about an institution, informed by institutional actions, and an antecedent of a more durable attitudes of trust, mistrust or distrust in that institution.

Measurement of trustworthiness is necessary for informing interventions to improve trustworthiness and begin to build community trust. This study, using data collected in the course of an internal evaluation conducted at an academic medical center, proposes an instrument to measure academic medical centers’ trustworthiness within their health care and research enterprises.

## Background

Research on trust in healthcare and academic health institutions has alternately conceived of the concept as trust in the clinicians, faculty and staff that operate within these institutions as well as trust in the institutions themselves [11,12]. Anderson and Griffith define trust in institutions as the extent to which individuals interacting with the institution perceive that its interests agree with their own interests [4]. Historically, assessing trust has relied on measuring it in two different ways: as an attitude or perception of the institution (affective trust) or as behavior (enacted trust) [11]. However, these two areas of research are largely siloed within disciplinary boundaries, and thus have not been integrated into a comprehensive theory of trust or a strategy for its measurement.

It is recognized though that trust in healthcare and academic health institutions cannot be built if there is a general perception of the institution or the individuals representing the institution and providing care or conducting research as untrustworthy. As a result, trust is often used in the empirical literature as a proxy for trustworthiness [4]. But, as a necessary antecedent for establishing trust in healthcare and academic health institutions, it is vital to understand and measure trustworthiness as a distinct concept [4]. By focusing on trustworthiness rather than trust, responsibility for trust is placed on institutions and the extent to which they have fostered the conditions that are necessary for trust [11,12]. This suggests the need for intervention research that promotes institutional change that would facilitate the cultivation of conditions that will foster community trust in that institution.

Intervention research of this type has been stymied by the lack of conceptual clarity that characterizes trustworthiness. It is not possible to directly observe trustworthiness, unlike affective or enacted trust [11–13]. The idea of trustworthiness implies a feeling or attitude based on a perception of how an (anthropomorphized) institution might behave in the future to represent one’s own interests. As Taylor and colleagues note, “The best we can do is query people about their subjective perceptions of trustworthiness – or willingness to trust” [11]. Further, trustworthiness, as a construct, is highly sensitive to context and how an individual assesses a particular context. Although researchers would prefer to understand trustworthiness over the long-term, the way that an individual assesses trustworthiness might change dramatically in a short span of time or depend on a recent experience with an institution. Because trustworthiness is dynamic and fundamentally perceptual in nature, it has proved challenging for researchers and interventionists to agree upon strict definitions of the concept.

Finally, trustworthiness has been difficult to study because of the disconnect between theory regarding the concept and its measurement. In the theoretical literature, trustworthiness is considered to be multifaceted and multilevel. However, empirical operationalizations of the concept have favored one-item, unidimensional scales, or proxy concepts (i.e., trust) to capture trustworthiness. These strategies have garnered criticism for a number of reasons. Single items are efficient, but simplistic. Single item measurement of trustworthiness is well-suited for contexts in which it is important to minimize respondent burden or to obtain a high-level picture of the opinion of a large group of people, such as in polling. Unidimensional scales are more useful in that they can capture more facets of trustworthiness and often undergo validation processes. Researchers have identified multiple dimensions of trustworthiness that are not sufficiently operationalized through unidimensional scales [12,14]. To assess trustworthiness in the context of institutional quality improvement or developing and assessing interventions to increase institutional trustworthiness, it is vital to engage in multidimensional operationalization of trustworthiness [12].

To our knowledge, there is one multidimensional scale of trustworthiness, the Perceptions of Research Trustworthiness (PoRT) scale [15]. This scale was designed to measure trust in biomedical research by members of minoritized racial and ethnic groups. It exemplifies the tendency to conflate the interrelated but distinct concepts of trustworthiness, trust, and distrust. The two dimensions that are used to capture “trustworthiness” in this scale are “trust” and “distrust.” McCullough and colleagues also propose a multidimensional conception of trustworthiness predicated on different types of trust: intellectual trust and moral trust [16]. Other theoretical work has identified a number of dimensions of trustworthiness, separate from trust. In particular, Mayer and colleagues identify three key elements of organizational trustworthiness: perception of ability/competence, benevolence, and integrity [17,18]. Complementary political trust literature emphasizes overall confidence in institutions as a multi-dimensional construct with dimensions of fairness and transparency added to competence and integrity [19]. Although empirical evidence has supported the importance of these dimensions of trustworthiness, research also suggests that these characteristics might be more relevant in determining interpersonal, rather than organizational trustworthiness [20].

Other work has sought to delineate principles of trustworthiness, specifically among healthcare institutions. In the interest of guiding healthcare and public health organizations in the process of building trust among community members they serve, the Association of American Medical Colleges (AAMC) published 10 Principles of Trustworthiness based on local perspectives and precepts of community engagement [21]. In developing these principles, the AAMC Center for Health Justice suggests that these are necessary foundations for a healthcare or academic health institution that seeks to engage community members in research. To facilitate institutions’ adoption of these principles in practical ways, AAMC also has provided a toolkit to guide interactions between institutions and communities in ways that will increase an institution’s trustworthiness and facilitate the building of trust. Given the declining trust in health institutions and the dearth of available and theoretically-grounded options, it is important to develop tools for systematically studying the antecedents of trust in academic health centers. Based on a commitment to integrating community perspectives in evaluating institutional trustworthiness and using the practices included in the reflection guides that are part of AAMC’s Principles of Trustworthiness Toolkit, we propose and psychometrically evaluate a 20-item multidimensional scale of institutional trustworthiness, the Multidimensional Institutional Trustworthiness Scale (MITS). Although this instrument was developed in the context of an internal organizational evaluation process, we believe that it can be used more broadly. We intend for this instrument to be used not just for internal quality improvement, but also to advance research on trustworthiness and to evaluate the effectiveness of interventions that seek to improve institutions’ trustworthiness.

## Methods

### Measure development

Inspired by the AAMC focus on trustworthiness in their community engagement toolkit, Anderson and Griffith’s conceptual framework for measurement, and the extant literature on community trust in community-engaged research from the national Engage for Equity team [10], we developed a 20-item measure to assess trustworthiness. This measure was developed as a part of a larger project to evaluate the community engagement actions within academic medical centers [22,23].

Four key domains were considered for the scale and included: (1) communication and coordination (8 items), (2) planning and action (6 items), (3) perceived benefit of community engagement (3 items), and (4) sustainable engagement (3 items). Specifically, the development of these items was informed by the reflection guides and interview and focus group guides associated with AAMC’s Principles of Trustworthiness [21,24,25], highlighting how individuals with institutional affiliations may perceive that institution’s trustworthiness. These questions were reviewed by the institutional team members engaged in the evaluation process, described elsewhere, who themselves were experts in community-based participatory research and co-authors on this paper. Because this was a mixed methods evaluation, several project team members drew on the qualitative findings (not presented here) to support the refinement of the survey items.

In communication and coordination, items queried about communications around the organization-wide policies when working with the communities, the institution’s ability to demonstrate an understanding that community engagement is valuable, the institution’s ability to support honest communications, and create an open, honest, and accessible environment for community members. Under the second domain – planning and action, items queried about the institution taking time to understand local knowledge and history, providing concrete ways in which researchers can incorporate community work, and its ability to follow-up on their words with meaningful actions. In the third domain of perceived benefit of community engagement, items queried the institutional researcher’s ability to consider diversity, value multiple identities, and meet and interact with community members where they work and live.

We include items regarding the behavior of individual researchers within the institution in recognition of the crucial role of institutional researchers as actors that mediate between community members and the institution. The degree to which individual researchers’ behavior is perceived as trustworthy or aligned with community interests can reflect the conditions within and priorities of the institution or system of which they are a part [4]. The fourth domain – sustainable engagement queried about the institutional ability to build and maintain trust within the community, incorporate dissemination plans and maintain ways to engage with community beyond a specific project.

All items in each domain reflect statements to which survey respondents would provide the degree to which they agreed with the statement using 5 Likert responses (“Strongly disagree,” “Disagree,” “Neither agree nor disagree,” “Agree,” and “Strongly agree”). When participants responded “Don’t know” or did not record a response, these responses were treated as missing data.

### Participants

Invitations to complete an online survey in REDCap were sent to 175 individuals affiliated with an academic medical center with 3 regional sites and one multi-state health system. Invitations were sent to individuals within the academic medical center who were in leadership positions, affiliated with the center’s clinical and translational science center, or who had experience conducting or contributing to community-engaged research (CEnR). Community partners were invited to participate if they had been involved in CEnR or served on a community advisory board with the academic medical center. A total of 5 rounds of invitations were sent between September 20, 2023, and October 6, 2023. We received 85 total survey responses, for a response rate of 49%; however, 79 of those participants (46 community partners and 29 academic medical center affiliates) responded to survey items related to trustworthiness. For further detail, our recruitment procedure and the broader survey instrument are described elsewhere [22,23].

Our sample had more self-identified community members (60%) compared to academic medical center affiliates (40%). Most respondents identified their race as White (54.1%) while 36.5% identified as Black or African American. Ethnicity was assessed separately, and 16.5% of respondents identified as Hispanic or Latino. Additionally, 62.4% of respondents identified as female, while 34.1% identified as male and 3.5% identified with another identity or preferred not to say.

### Analysis plan

Descriptive statistics were computed for all 20 items on the institutional trustworthiness scale. For each item, we examined response distributions, including the percentage of missing data, means, standard deviations, minimum and maximum values, and skewness. These indicators were recommended by Petrillo and colleagues to evaluate the quality of the data and the appropriateness of the response scale, which ranged from 1 (“Strongly Disagree”) to 5 (“Strongly Agree”) for this instrument [26]. Ideally, participants would utilize the full range of the scale to capture a broad representation across the four domains.

**Figure 1.**
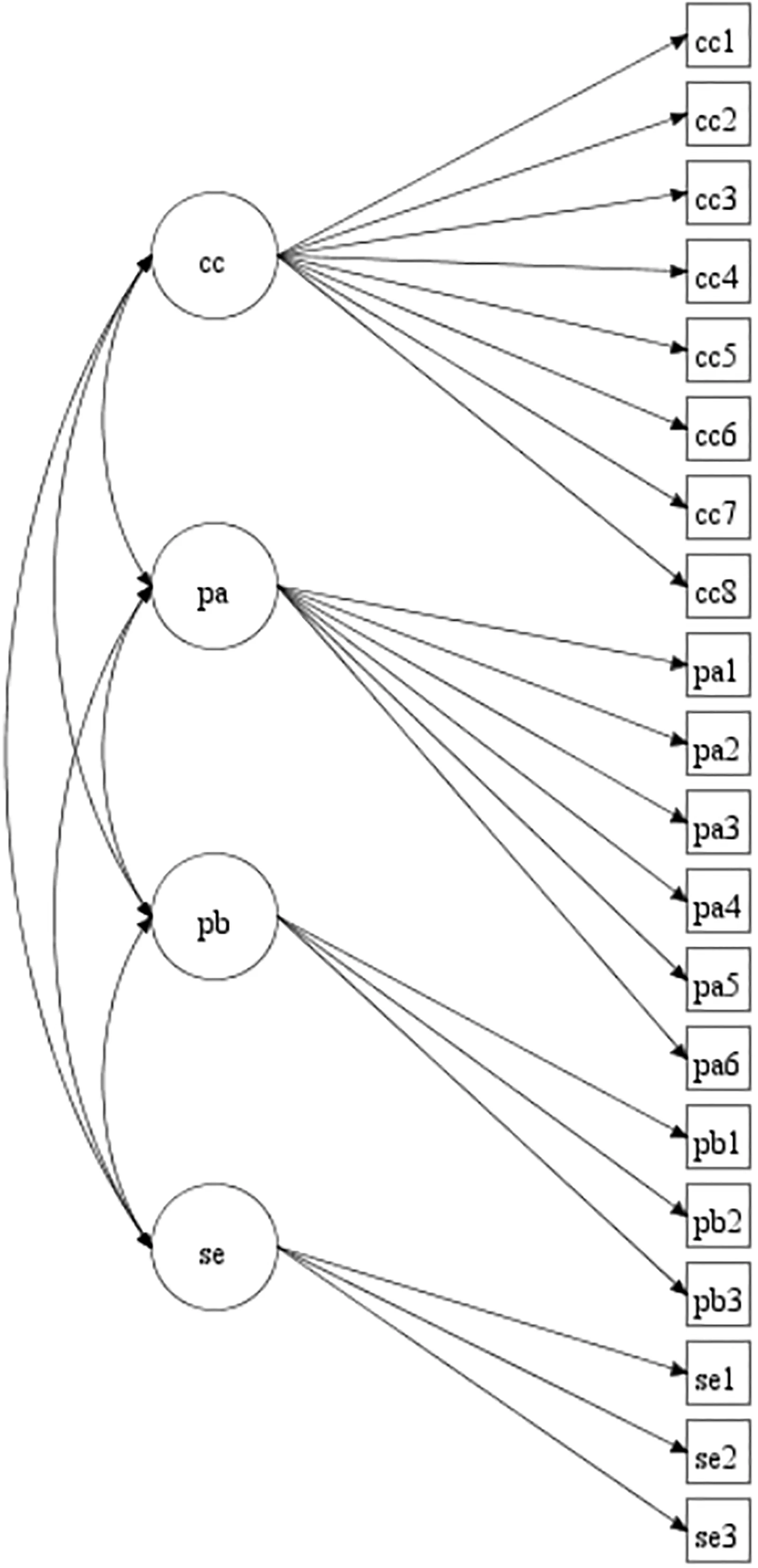
Figure 1 long description. The current model showed adequate model fit: chi-square (164) = 280.993, *p* = <0.001; RMSEA = 0.096; 90%CI of RMSEA = [0.076, 0.114]; CFI = 0.916; TLI = 0.903; SRMR = 0.052.

Next, item-total correlations were calculated to assess the coherence between individual items and their respective subscale scores. Item-scale correlations exceeding 0.30 were considered acceptable, indicating that items were appropriately grouped within the same factor. To further evaluate the psychometric properties of the institutional trustworthiness scale, construct validity was assessed using confirmatory factor analysis (CFA). CFA is particularly suitable for evaluating theoretically-driven models in which item selection is guided by a conceptual framework. In this study, we tested a four-factor model aligned with the conceptualization of institutional trustworthiness in the MITS, consisting of the following latent constructs: communication and coordination (CC), planning and action (PA), perceived benefit of CEnR (PB), and sustainable engagement (SE) (see Figure 1).

Model fit was assessed using the chi-square statistic and three commonly used fit indices: the Comparative Fit Index (CFI), the Root Mean Square Error of Approximation (RMSEA), and the Standardized Root Mean Square Residual (SRMR). Model fit was considered acceptable if RMSEA < 0.10, CFI > 0.90, and SRMR < 0.10 [27–29]. Modification indices (MIs) were reviewed to inform potential model re-specifications aimed at improving model fit. After finalizing the model, standardized factor loadings were reported to reflect the strength of the relationship between each item and its corresponding latent factor. Correlations among latent factors were also reported. If factor correlations exceeded 0.70 – indicating potential conceptual overlap – we tested a one-factor model and compared its fit to that of the original four-factor model. We only tested a four-factor model and a one-factor model, and avoided testing other possible factor structures. The four-factor model was hypothesized based on existing literature. We tested a one-factor model to determine whether a unidimensional measure of trustworthiness was supported by the data [23].

We adopted a holistic approach to item evaluation. Items identified as underperforming – based on both poor descriptive properties and factor loadings below 0.40 – were considered for removal from the final scale. Once the factor structure and item set were finalized, scale score reliability was assessed. Internal consistency was estimated using Cronbach’s alpha, and composite reliability was calculated using omega coefficients [30].

All descriptive statistics, Cronbach’s alpha coefficients, and Pearson’s correlations were conducted using SPSS (Version 28) [31]. Omega composite reliability values were computed using the R package *MBESS* [32]. CFA tests were performed using Mplus Version 8.7 [33].

## Results

### Descriptive statistics

Demographic description of the sample is provided elsewhere [23]. Descriptive statistics for the 20 items are presented in Table 1. All items had observed responses spanning the full range of the 5-point Likert scale (1 to 5), indicating that participants used the entire response spectrum to express their perceptions of institutional trustworthiness. Among the 79 participants who responded to the trustworthiness items, item-level missing data (no responses and “don’t know” responses combined) ranged from 1 to 16%. Skewness values ranged from −0.95 to −0.09, and kurtosis values ranged from −1.22 to 1.22, suggesting that the item responses approximated normal distributions [30]. Mean scores across the items ranged from 3.12 to 3.93, indicating generally favorable perceptions of institutional trustworthiness among participants. Item-scale correlations ranged from 0.69 to 0.88, providing preliminary evidence of coherence among the items.

**Table 1.** Item wise performance of the scale (*n* = 79) Table 1 long description.

Item	na	DK	Min.	Max.	Mean	SD	Skewness	Item-scale correlations
Communication and coordination								
[Institutional] researchers communicate organization-wide policies and expectations when working with the community (CC1)	1	7	1	5	3.68	1.05	−0.44	0.77
[The institution] demonstrates an understanding across the organization that community engagement in research is valuable (CC2)	1	3	1	5	3.37	1.29	−0.20	0.79
[The institution] demonstrates a commitment to adequately compensate community partners for their work (CC3)	0	5	1	5	3.53	1.11	−0.32	0.69
[Institutional] researchers support honest communication with the community (CC4)	0	3	1	5	3.91	0.93	−0.95	0.81
The [institution] routinely engages community organizations and community leaders to assess potential new partnerships (CC5)	0	1	1	5	3.74	0.96	−0.55	0.81
[Institutional] researchers demonstrate clarity and intentionality about working with the community (CC6)	0	2	1	5	3.68	1.13	−0.56	0.88
[Institutional] researchers co-develop expectations and goals with the community (CC7)	0	9	1	5	3.44	1.11	−0.24	0.88
[Institutional] researchers create an open, honest, and accessible environment where community members feel safe and are able to provide feedback (CC8)	1	3	1	5	3.69	0.99	−0.47	0.81
Planning and action								
[Institutional] researchers have taken the time to understand local knowledge and history of the communities with whom they work (PA1)	3	3	1	5	3.32	1.14	−0.19	0.83
There are concrete ways in which [institutional] researchers have incorporated community expertise in their work (PA2)	3	1	1	5	3.79	0.93	−0.88	0.76
[The institution’s] strategy reflects how CEnR aligns with the assets, needs, and goals of the community (PA3)	3	3	1	5	3.30	1.14	−0.16	0.80
[Institutional] researchers follow up on their words with meaningful action (PA4)	3	3	1	5	3.58	1.12	−0.50	0.76
[Institutional] plan for action often holds the organizational leadership accountable (PA5)	3	10	1	5	3.12	1.12	−0.18	0.84
[The institution’s] plan for action is co-developed with the community (PA 6)	3	7	1	5	3.23	1.13	−0.09	0.83
Perceived benefit of community-engaged research (CEnR)								
[Institutional] researchers consider that diversity is more than race and ethnicity (PB1)	4	4	1	5	3.93	0.96	−0.95	0.70
[Institutional] researchers understand and value the multiple identities and lived experiences of community members (PB2)	3	5	1	5	3.79	1.05	−0.84	0.80
[Institutional] researchers often meet and interact with community members where they work and live. (PB3)	3	6	1	5	3.50	1.10	−0.57	0.72
Sustainable engagement								
[Institutional] researchers continue to build and maintain trust within the community (SE1)	4	4	1	5	3.79	0.96	−0.98	0.86
Evaluation and dissemination plans are built into partnerships with community members (SE2)	5	8	1	5	3.59	1.10	−0.57	0.83
[Institutional] researchers find ways to engage community partners when not tied to a specific project (SE3)	5	4	1	5	3.36	1.22	−0.43	0.79

*Note*: na = did not record a response; DK = “don’t know” response.

### Construct validity

To evaluate the construct validity of the institutional trustworthiness scale, CFA was conducted based on the hypothesized four-factor structure. Given the approximately normal distribution of item responses, we employed maximum likelihood estimation under the assumption that data were missing at random. Modification indices were reviewed to guide model refinement [30].

The four-factor model demonstrated acceptable fit to the data: χ^2^(164) = 280.99, *p* < 0.001; RMSEA = 0.096, 90% CI [0.076, 0.114]; CFI = 0.916; TLI = 0.903; SRMR = 0.052. Correlations among the four latent factors – Communication and coordination (CC), Planning and Action (PA), perceived benefit of CEnR (PB), and Sustainable Engagement (SE) – were high, ranging from 0.91 to 0.96. Given this level of overlap, we tested an alternative one-factor model. A chi-square difference test indicated that the four-factor model fit the data significantly better than the one-factor model, Δχ^2^(6) = 41.99, *p* < 0.001, supporting the multidimensional structure of institutional trustworthiness.

Standardized factor loadings for all items exceeded 0.70, with the exception of one item “[The academic medical center] demonstrates a commitment to adequately compensate community partners for their work” from the Communication and Coordination domain, which had a loading of 0.65 – still well above the conventional threshold for item retention. Together, these results indicate that the hypothesized factors explained a substantial portion of item variance (typically > 50%). No item was flagged for removal based on either its factor loading or descriptive statistics. A sensitivity analysis was conducted to compare the model fit indices between the primary model where “don’t know” responses treated as missing and a model where such responses were recoded as the scale midpoint (Model_*mid*_). While both models demonstrated adequate fit, Model_*mid*_ showed a marginal improvement in indices (e.g., CFI increased by 0.019 and RMSEA decreased by 0.013). This suggests that for this specific population, uncertainty may function similarly to a neutral evaluation, though the structural relations between factors remained consistent across both methods. In sum, findings from the CFA provide strong empirical support for the proposed four-factor model of institutional trustworthiness, affirming its conceptual structure and measurement integrity.

### Scale reliability

Internal consistency reliability was assessed for each subscale. Cronbach’s alpha values were 0.95 for communication and coordination, 0.93 for planning and action, 0.86 for perceived benefit of CEnR, and 0.91 for sustainable engagement, indicating excellent internal consistency. Composite reliability, estimated using omega coefficients, yielded values of 0.95 for communication and coordination, 0.93 for planning and action, 0.87 for PB of CEnR, and 0.92 for SE. These reliability estimates suggest that all four subscales produce consistent and dependable scores, supporting their use for assessing individual differences in institutional trustworthiness [34].

## Discussion

This study presents the development of a novel multidimensional scale for assessing institutional trustworthiness that was informed by the AAMC toolkit. These survey items were categorized under four domains: communication and coordination, planning and action, perceived benefit of community-engaged research (CEnR), and sustainable engagement, representing the antecedent conditions that contribute to individual’s perceptions of institutional trustworthiness. Psychometric testing of this 20-item survey supports the construct validity of this multidimensional four factor model in an initial sample of 79 respondents affiliated with an academic medical center. These respondents were a mix of community members who were familiar with the institution either with direct healthcare experience or engagement in a research project or an advisory board; as well as academic leaders, investigators, and staff. The development and psychometric testing of this scale allows for future accurate and multidimensional assessments of institutional trustworthiness that can inform the development of interventions and strategies [35].

**Figure 2.**
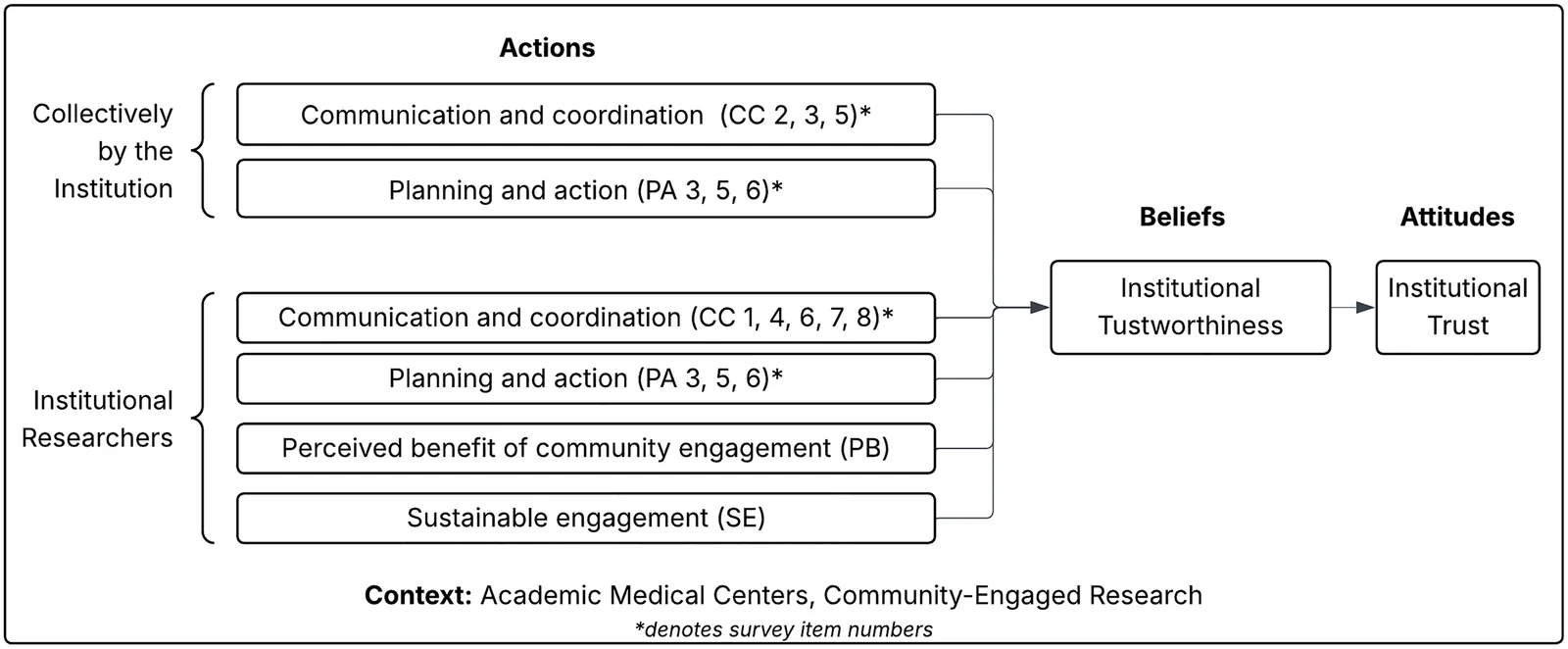
Conceptual model linking institutional and institutional researchers’ actions to attitudes of institutional trust through perceived beliefs about institutional trustworthiness in community-engaged research.

The Multidimensional Institutional Trustworthiness scale was created as a part of an evaluation project and helps delineate how institutional and institutional researcher actions shape trust in academic medical centers where community-academic partnerships are leading community-engaged research (See Figure 2). At the institutional level, collective actions around communication, coordination, and planning reflect organizational structures and processes that signal responsiveness and alignment with community priorities. At the level of institutional researchers, actions encompass communication, coordination, and planning, perceived benefit of community engagement, and effort undertaken towards sustainable engagement, which represent how research teams operationalize engagement in practice.

These actions converge to influence beliefs about institutional trustworthiness, which in turn, shape institutional trust that is conceptualized as a downstream attitudinal orientation, consistent with the Anderson and Griffith model. By distinguishing trustworthiness as comprised of beliefs about institutional actions from trust as an attitude, this model contributes to the literature by positioning trustworthiness as an emergent outcome of multilevel actions, derived from the toolkits created to support the principles of trustworthiness by AAMC. This framework is situated within academic medical centers and particularly salient for community-engaged research, where trust is foundational to partnership.

It is important to note that this measure was developed as an attempt to conceptually distinguish perceptions about trustworthiness from trust. In other words, we were concerned with identifying the characteristics and actions of institutions that made them perceived as trustworthy. This measure reflects the degree to which an institution demonstrated actions characterized by consistent communication and coordination, planning and action, positive perceptions of community engagement, and sustaining relationships. Perception of these institutional actions supporting it to be trustworthy among the individuals it employs and serves. Trustworthiness is reflected in how individuals perceive the attributes and actions of institutions. Trustworthiness must be actively cultivated through accountable actions and policies.

Trustworthiness therefore is not a static trait or attribute [20,36]. It must be demonstrated consistently, especially in response to evolving community needs, historical injustices, and societal changes. Ongoing, longitudinal assessments with a reliable scoring system and valid score interpretations are therefore critical to help identify gaps between institutional intent and impact, allowing for timely course corrections and continued improvement. Longitudinal assessments can also help build accountability, showing communities that institutions are committed to action.

Study findings also support treating trustworthiness as a multi-factor model for assessment as opposed to single-factor models that have been described in the literature. Trustworthiness is a multidimensional construct that encompasses several actions and attributes that collectively and uniquely contribute to how individuals with differing relationships to the institutions might perceive. A single-factor model could oversimplify this complexity and miss critical areas. By contrast, a multifactor model can offer a more targeted assessment. In this study, all items performed well and the four-factor model outperforms the one-factor model.

There are limitations to this work. Our study relied on a relatively small sample size (*n* = 79) for the confirmatory factor analysis. Conventional guidelines often recommend larger samples; however, the impact of sample size on model stability is highly dependent on factor overdetermination and the magnitude of indicator loadings [37]. In the present study, the model benefited from high overdetermination (on average five indicators per factor) and robust factor loadings (>0.70), which mitigate the risks of parameter bias and non-convergence typically associated with smaller samples [38]. Furthermore, the high inter-factor correlations (ranging from 0.91 to 0.96) provided additional structural information that stabilizes the model estimation at lower participant counts. Nevertheless, while these factors support the internal structure of the current findings, the small sample size may limit the generalizability of the results. Future research should seek to replicate these findings in larger, more diverse cohorts to confirm the stability of the four-factor structure across different populations and to investigate the reliability and validity of this measure for different use cases (e.g., research, quality improvement).

Additionally, there were 8 items where more than 10% of responses were “Don’t know” or missing. This potentially reflects what existing literature postulates as a lack of conceptual clarity around operationalizations of the dimensions of trustworthiness. Future research should strive to refine these domains and substantiate their generalizability to broader populations and in different organizational contexts. This high representation of “Don’t know” and missing responses may also reflect respondents’ subjectivity and lived experience based on their relationship with the institution, which might impact their ability to know about the specific actions and policies that are undertaken by the institution. For example, community members that are engaged with a specific research project may have a narrow experience with the institution, and their perception of the trustworthiness of the institution might be swayed heavily by their (positive or negative) interactions with individual institutional researchers. Among community members for whom this is the case, there is likely to be substantial variation in experiences with individuals from the institution, depending on the level of experience a given researcher has with engaging community members [23,39].

And although the model fit is adequate, in the sensitivity analysis for the CFA results, the presence of “don’t know” responses suggests that the institution has yet to provide the necessary insights for all stakeholders to form a substantive perception of trustworthiness. Our sample did not allow us to begin to tease apart these differences. Although it is important for future work with this measure to strive for larger sample sizes to investigate these differences, our sampling strategy did seek to solicit responses from participants with history with the institution. It is possible that there was substantial variation both within and between community and academic participants in their perceptions of the institution.

### Future directions

This study provides psychometric evaluation for a measure developed for a specific context – assessing the academic medical center’s trustworthiness from both an academic and community member perspective. In this context, our results support the reliability of the scale scores and provide evidence to support the interpretation of scores derived from the measure, showcasing a four-factor model that assessed the institution’s trustworthiness. This represents one specific way to assess trustworthiness, through the institutional and researcher actions, and should not be considered as the only approach to such an assessment.

Complementary approaches such as qualitative interviews, listening sessions, and group-based modeling, could supplement surveys and provide a more thorough and accurate understanding of this construct for institutions. Future research should look at additional variables such as institutional credibility and reputations, transparency, and responsiveness, as additional factors that could deepen our understanding of institutional trustworthiness. Additionally, future research should seek to recruit larger samples of participants, both community members and institutional actors, to disentangle differences in the way that the MITS operates for these two populations. In addition, community partners’ reviews and cognitive interviews would also help further refine the survey items. In particular, measurement invariance testing to assess whether these subscales operate comparably across these groups would be extremely valuable. Further, it would be important to incorporate the perceptions of patients and more diverse groups of community members (not just those with experience participating in research or research activities with the institution). Future scale validation efforts should involve investigating the instrument’s use in other healthcare or academic health institutions as well as for different goals (e.g., research, evaluation, quality improvement).

Given declining trust in health institutions and institutions in general, future work should conduct longitudinal assessments and additional reliability checks to assess the value of the scale in measuring perceptions of institutions and intervention development. Consistent measurement of perceptions of institutional trustworthiness and concomitant evaluation of these measurement tools would provide insights into the mechanisms that promote perceptions of trustworthiness, ultimately leading to improved trust in health care providers and research at academic medical centers. Institutional level interventions for strengthening practices and policies related to CEnR are currently being explored [39–41], but more interventions with the explicit goal of promoting trustworthiness need to be implemented and evaluated. By establishing and examining the psychometric properties of this multidimensional tool, this research offers a preliminary step in developing interventions to address these mechanisms and promote institutional trustworthiness in an era when public trust in health institutions is particularly low.

## Figure 1. Long description

A diagram illustrating the relationships between different components of trust in healthcare and health research institutions. Panel A: The diagram shows a flowchart with four main components labeled cc, pa, pb, and se. Each of these components is connected to several sub-components. The component cc is connected to sub-components cc1 through cc8. The component pa is connected to sub-components pa1 through pa6 and pb1 through pb3. The component pb is connected to sub-components pb1 through pb3. The component se is connected to sub-components se1 through se3. Arrows indicate the direction of influence or relationship between the main components and their sub-components. Panel B: The diagram does not include any specific values, labels, or axes. The focus is on the structural relationships and the flow of influence between the components.

Navigate back to Figure 1..

## Table 1. Long description

A table with 20 rows and 9 columns detailing the performance of a scale with 79 participants. The columns are labeled Item, na, DK, Min., Max., Mean, SD, Skewness, and Item-scale correlations. The table is divided into four sections: Communication and coordination, Planning and action, Perceived benefit of community-engaged research, and Sustainable engagement. Each row represents an item with corresponding statistics. For example, Row 1: Institutional researchers communicate organization-wide policies and expectations when working with the community (CC1), na: 1, DK: 7, Min.: 1, Max.: 5, Mean: 3.68, SD: 1.05, Skewness: -0.44, Item-scale correlations: 0.77. Row 2: The institution demonstrates an understanding across the organization that community engagement in research is valuable (CC2), na: 1, DK: 3, Min.: 1, Max.: 5, Mean: 3.37, SD: 1.29, Skewness: -0.20, Item-scale correlations: 0.79. The table provides detailed statistics for each item, including the range of responses, mean scores, standard deviations, skewness, and item-scale correlations.

Navigate back to Table 1..
